# The Influence of Energy Depletion by Metformin or Hypocaloric Diet on Thyroid Iodine Uptake in Healthy Volunteers: a Randomized Trial

**DOI:** 10.1038/s41598-019-41997-2

**Published:** 2019-04-01

**Authors:** Yvette J. E. Sloot, Marcel J. R. Janssen, Antonius E. van Herwaarden, Robin P. Peeters, Romana T. Netea-Maier, Johannes W. A. Smit

**Affiliations:** 1 Department of Internal Medicine, Division of Endocrinology, Radboud University Medical Center, Geert Grooteplein Zuid 8, 6525 GA Nijmegen, The Netherlands; 2 Department of Radiology and Nuclear Medicine, Radboud University Medical Center, Geert Grooteplein Zuid 8, 6525 GA Nijmegen, The Netherlands; 3 Department of Laboratory Medicine, Radboud University Medical Center, Geert Grooteplein Zuid 8, 6525 GA Nijmegen, The Netherlands; 4 Department of Internal Medicine, Academic centre for Thyroid Diseases, Erasmus University Medical Center, Dr. Molewaterplein 40, 3015 GD Rotterdam, The Netherlands

## Abstract

Sufficient thyroid iodine uptake is needed to ensure effective radioactive iodine (RAI) treatment, which is mediated by the sodium-iodide symporter (NIS). Activation of AMP-activated-protein-kinase (AMPK), leads to decreased NIS expression and thyroid iodine uptake in *in vitro* and animal models. Clinically relevant conditions that lead to AMPK activation include metformin use and hypocaloric conditions. Here, we aim to assess the effects of metformin and hypocaloric diet on thyroid iodine uptake in healthy volunteers. Healthy male volunteers were included and randomized. Group 1 (n = 8) received metformin, group 2 (n = 7) followed a hypocaloric diet (1500 kcal/day), superposed on a moderate iodine restriction diet; Baseline measurements included thyroid iodine-123 (I-123) uptake and TSH, fT4, T3 and rT3 levels. After two weeks, thyroid function and I-123 uptake measurements were repeated. Baseline characteristics were similar between groups. Levels of TSH and fT4 were similar after each intervention. T3 decreased after hypocaloric diet and metformin (−0.2 ± 0.19 nmol/L, p = 0.0327; respectively −0.13 ± 0.13 nmol/L, p = 0.0282), resulting in decreased T3/rT3 ratios. There was no significant difference in thyroid I-123 uptake after each intervention. In conclusion, metformin treatment and hypocaloric diet resulted in a significant decrease in T3 levels and T3/rT3 ratios in healthy volunteers, without significant effects on thyroid iodine uptake. We found no indications that metformin or hypocaloric diet will have clinically relevant effects on RAI uptake.

## Introduction

Treatment with radioactive iodine (RAI) is a widely used and highly efficient treatment for benign thyroid diseases and differentiated thyroid carcinoma (DTC)^1,2^. To improve RAI treatment efficacy and reduce potential side effects, it is important to identify factors that influence thyroid RAI uptake.

The specific thyroid iodine uptake is mediated by the sodium iodide symporter (NIS)^3^. The main regulator of NIS expression is thyroid stimulating hormone (TSH), but other regulatory pathways have been described as well^4,5^. Previous research has shown that activation of the master sensor for energy depletion, 5′ adenosine monophosphate-activated-protein-kinase (AMPK), leads to decreased NIS expression and iodine uptake in *in vitro* studies and animal models^6–8^. Conditions that deplete cellular energy stores induce activation of AMPK, which leads to downregulation of energy consuming processes and upregulation of adenosine triphosphate (ATP)-producing pathways in order to restore the cell’s energy balance^9^.

In rat thyroid cell lines and in animal studies, activation of AMPK using metformin or 5-aminoimidazole-4-carboxamide ribonucleotide (AICAR) significantly reduced NIS expression and iodine uptake through reduced transcription and increased degradation of NIS^6–8^. These results show that modulation of AMPK activity influences NIS expression and RAI uptake.

Metformin is a first-choice drug in type 2 diabetes mellitus (DM)^10^ and a well-known pharmacological modulator of AMPK^11^. Metformin is being investigated as adjunct in the treatment of DTC^12^, as its use has been associated with beneficial effects on DTC in preclinical and patient studies^13–15^. However, metformin has also been shown to activate AMPK leading to reduced NIS expression *in vitro* and in animal models^6^. Metformin may therefore reduce RAI therapy efficacy.

Reduced caloric intake also leads to AMPK activation, and could thus also result in reduced NIS expression and RAI uptake^9^. Preparation for RAI treatment in DTC consists of either thyroid hormone withdrawal for 3–4 weeks or administration of recombinant human TSH (rhTSH) in order to stimulate thyroid iodine uptake^2^. Thyroid hormone withdrawal is associated with symptomatic hypothyroidism and reduced caloric intake due to appetite loss, fatigue, weight changes, gastro-intestinal complaints and depression^16,17^. In addition, an iodine depleted diet which is often used in preparation for RAI therapy, is also accompanied by decreased appetite and caloric intake^18^.

Energy depletion by metformin and hypocaloric conditions are thus clinically relevant conditions that lead to AMPK activation, and we hypothesize that these could result in reduced thyroid iodine uptake. Therefore, in this study we aim to assess the physiological effects of energy depletion by metformin and hypocaloric diet on thyroid iodine uptake in healthy volunteers.

## Results

### Baseline characteristics

A total of 29 volunteers were screened and had provided signed informed consent. Nine subjects withdrew before the start of the study. One subject started with the study but was not able to comply with the iodine restriction and left the study. For our analysis, we included a total of 19 healthy male volunteers between the ages of 19 and 43 years, who completed the study, with a mean age of 24.7 ± 5.5 years. Baseline parameters (Table 1) were comparable between the two intervention groups, with exception of a slightly higher self-reported daily energy intake in the hypocaloric diet group (3082 ± 390 kcal/day), compared to the metformin group (2404 ± 305 kcal/day, *p* = *0*.*008*).

**Table 1:** Baseline characteristics of the three intervention groups.

	Total	Diet	Metformin
	n = 19	n = 7	n = 8
Age	24.7 (5.5)	24.7 (2.6)	26.3 (7.9)
Height (m)	1.8 (0.1)	1.9 (0.1)	1.8 (0.1)
Weight (kg)	76.6 (8.7)	79.1 (11.7)	73.7 (6.9)
BMI (kg/m2)	22.9 (2.0)	22.8 (2.4)	22.6 (2.2)
Daily resting Energy Need (Kcal)^§1^	1805 (225)	1778 (352)	1793 (129)
Activity factor (PAL)	1.7 (0.1)	1.6 (0.2)	1.6 (0.1)
Total Energy Expenditure (Kcal)^¶^	3006 (503)	2946 (748)	2931 (329)
Lean Body Mass (kg)^†^	57.6 (4.8)	59.6 (6.2)	55.9 (4.0)
Mean daily intake (Kcal)	2704 (466)	3082 (390)*	2404 (305)

Notes: Values are presented as mean ± S.D.

*Comparison between hypocaloric diet group and metformin group p = 0.008;

^§^Calculated with Harris and Benedict equation (ref.^36^ for men. Daily resting Energy Need = 88.362 + (13.398 × weight (kg)) + (4.799 × length (cm))− (5.677 × age).

^¶^Total Energy Expenditure = Resting energy need × PAL.

^†^According to formula of Hume (ref.^37^). Lean body mass = (0.32810 × weight (kg)) + (0.33929 × length (cm))−29.5336.

Subjects were compliant with the dietary restrictions of the hypocaloric diet. Mean total weight loss after intervention was 1.8 ± 2.4 kg in the diet group, and 0.8 ± 1.4 kg for the metformin group. BMI in the diet group decreased from 22.8 ± 2.4 kg/m^2^ to 22.2 ± 2.3 kg/m^2^ (*p* = *0*.*077*), whereas the decrease in the metformin group was 0.2 ± 0.4 kg/m^2^, see Supplementary Table 2.

Iodine excretion before interventions demonstrated the adherence of the participants to the low iodine diet. No significant differences were found in urinary iodine excretion between baseline and post intervention measurements (Supplementary Table 2).

Compliance to metformin was high; two subjects forgot one dose of metformin once. Apart from minor gastro-intestinal side effects, no major side effects of metformin were reported.

### Effect of metformin and hypocaloric dieting on TSH and thyroid hormone levels

**Figure 1:**
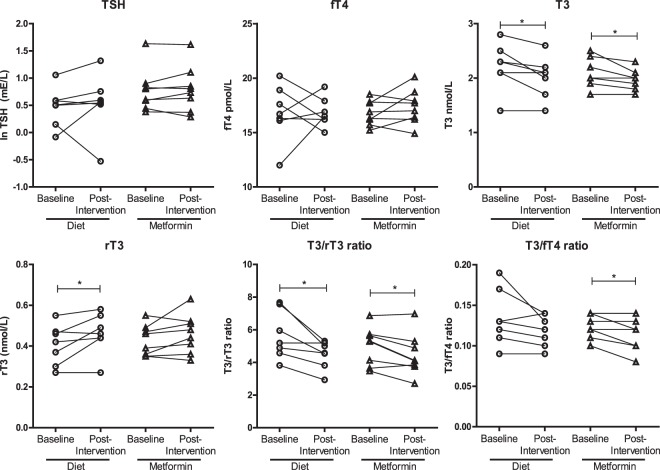
Effects of hypocaloric dieting and metformin on serum TSH, fT4, T3, rT3, the T3/rT3 ratio and T3/fT4 ratio in healthy volunteers before and after intervention. Serum TSH levels were log transformed and differences before and after intervention were determined with paired student’s T tests. *P < 0.05: significant effect of interventions within groups when compared with baseline.

Levels of s-TSH, s-fT4, s-T3 and s-rT3 at baseline and after each intervention are depicted in Fig. 1. There were no significant differences in s-TSH levels and s-fT4 levels before and after each intervention within each group, indicating that hypocaloric dieting or metformin did not affect TSH or fT4 levels. Serum levels of TSH and fT4 were also similar between the groups at baseline or after intervention. Interestingly, hypocaloric dieting and metformin lead to a significant decrease in s-T3 levels within the normal range, with a reduction of 0.2 ± 0.19 nmol/L (*p* = *0*.*033*), respectively 0.13 ± 0.13 nmol/L (*p* = *0*.*028*). The T3/fT4 ratio decreased after hypocaloric dieting from 0.134 ± 0.035 to 0.119 ± 0.020, and was significantly reduced after metformin use from 0.125 ± 0.015 at baseline to 0.114 ± 0.019 (*p* = *0*.*015*) post intervention. In addition, s-rT3 increased after two weeks of hypocaloric diet from 0.41 ± 0.10 nmol/L to 0.46 ± 0.10 nmol/L (*p* = *0*.*050*), resulting in a significant decrease of the T3/rT3 ratio of 5.7 ± 1.5 at baseline vs. 4.5 ± 0.8 post intervention (*p* = *0*.*019*). In the metformin group, a significant decrease of the T3/rT3 ratio from 5.0 ± 1.2 to 4.5 ± 1.3 was observed as well (*p* = *0*.*022*).

In the 4 control subjects, baseline measurements were within the normal range. As expected, no significant differences were observed in s-TSH, s-fT4 levels, s-T3 and s-rT3 levels during the course of the study.

### Effect of metformin and hypocaloric dieting on thyroid radioactive iodine uptake

**Figure 2:**
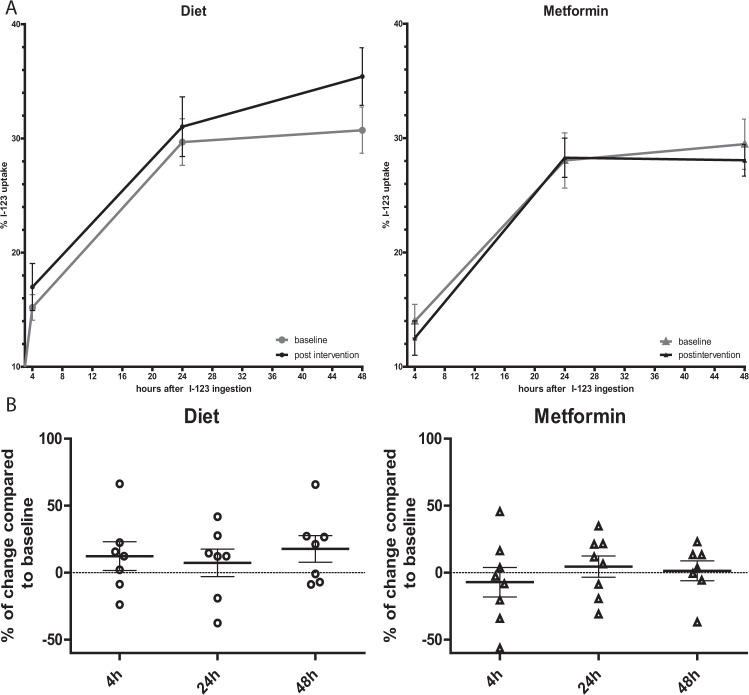
Effects of hypocaloric dieting and metformin on thyroid iodine-123 (I-123) uptake. Panel A shows the I-123 uptake at 4 hours, 24 hours and 48 hours after ingestion at baseline and after intervention. Panel B shows the percentage of change in I-123 uptake compared to baseline measurements at the three different time points for each intervention. Values are depicted as mean ± SEM.

To assess the effect of metformin use and hypocaloric dieting on thyroid iodine uptake, we measured thyroid I-123 uptake (Fig. 2A). There were no significant changes in I-123 uptake compared with baseline in any of the groups. In addition, I-123 uptake measurements were similar between groups, both at baseline and after intervention. We also calculated the percentage of change in I-123 uptake compared to baseline (Fig. 2B). There were no significant differences in percentage of change in I-123 uptake. These results indicate that hypocaloric dieting or metformin use did not result in significant changes in thyroid I-123 uptake in healthy volunteers.

## Discussion

To our knowledge, this is the first study investigating the effects of energy depletion by metformine or hypocaloric diet, both important AMPK activators, on thyroid iodine uptake in humans. We showed that hypocaloric dieting or metformin lead to a significant decrease in s-T3 levels and a significantly decreased T3/rT3 ratio whereas thyroid iodine uptake was not influenced.

A decreased T3/rT3 ratio after hypocaloric dieting as observed in our study is in line with other studies in which low energy diets are associated with a reduced ratio of T3/rT3^19–23^ by decreased deiodinase 2 (D2) and deiodinase 1 (D1) activity and an increase in deiodinase 3 activity (D3)^19–21,24^. Furthermore, selective carbohydrate deficiency specifically results in reduced peripheral conversion of T4 to T3 through reduced D2 activity^20,22^. Although prolonged weight loss has been associated with a reduction of s-TSH^19,25,26^, we did not observe significant changes in TSH levels after hypocaloric dieting, which is likely due to the fact that the observed weight loss was only small and the time of intervention was short.

Interestingly, in the metformin group we also observed a significant reduction of s-T3 levels, a decreased T3/rT3 ratio, slightly elevated s-rT3 levels and unchanged s-TSH and s-fT4 levels. These results suggest that metformin influences peripheral deiodination as well, as the use of serum T3/rT3 and T3/T4 ratios are validated parameters for deiodinase activity^27,28^. In contrast to our findings, a study from Krysiak *et al*. (2018) showed that treatment with metformin for several months did not affect TSH, fT4 and T3 levels in euthyroid patients with type 2 diabetes^29^. Possible explanations could be the difference in study population, the dose of metformin and the duration of the study. Insulin resistance and type 2 diabetes is associated with type 2 thyroid allostatic load, e.g. higher levels of active thyroid hormone T3 due to upregulation of peripheral deiodinases and increased plasma protein binding^30,31^. In contrast, conditions in our study, e.g. energy depletion by hypocaloric dieting and short-term metformin use in healthy volunteers, are examples of type 1 thyroid allostasis, resulting in downregulation of active thyroid hormone synthesis. Type 1 thyroid allostasis is associated with low or normal TSH and fT4 levels and a reduction in T3, caused by a downregulation of D1 and D2 deiodinases and decreased plasma protein binding of thyroid hormones^30^. Further research is necessary to investigate the short and long-term effects of metformin on T3 levels and deiodinase activity.

Our hypothesis that energy depletion by hypocaloric diet or metformine leads to decreased iodine uptake was not confirmed by our study. We postulate that this is related to the unchanged TSH levels. Although we were not able to perform thyroid tissue AMPK measurements, previous research suggests that caloric restriction similar to our study and even for a duration of only five days, effectively results in increased AMPK activity in other tissues^32^. Likewise metformin treatment in a clinically relevant dose as used in our study, results in AMPK activation *in vivo* as well^33^. Thus, although the fact that we were not able to study thyroid tissue AMPK is an important limitation, we believe that short term energy depletion does not lead to significant alterations in thyroid iodine uptake as TSH is not affected.

Limitations of this study include the short duration and the fact we only included a relatively small number of subjects. The short duration of the study was chosen by purpose, as the clinical setting of radioiodine therapy preparation involves a preparatory phase of several weeks. Although prolonged energy depletion could have given different results, we believe that the current design is more relevant for the context of radioiodine therapy. With respect to the number of participants, the study had sufficient power to detect subtle changes in thyroid hormone levels. Furthermore, this study would have 80% power to pick up a mean difference of ≥8.0% in I-123 uptake between baseline and post intervention, e.g. a mean reduction of ±26%. Indeed, previous *in vivo* studies in animals, showed a very large effect size with a reduction of thyroid I-uptake up to 35% after AICAR treatment as well^7^.

This mechanistic study was performed in healthy volunteers to enable unbiased analyses of study parameters. As aberrant AMPK activity, independent of energy availability, has also been associated with thyroid cancer^34^, we cannot extrapolate the study results to patients with benign thyroid disease and DTC. However, we believe that the fact that a short course of energy depletion does not affect endogenous TSH and thyroid iodine uptake, is relevant for clinical practice.

In conclusion, two weeks of metformin use or hypocaloric dieting decreased serum T3/rT3 ratios, but did not lead to significant and negative effects on radioactive iodine uptake in healthy volunteers. This may be relevant for radioiodine therapy in patients with thyroid disorders.

## Methods

### Subjects

Healthy male volunteers were recruited by advertisements on the website and digital student forums of the Radboud University Medical Center Nijmegen, and handing out advertisement flyers on several locations in the vicinity of the hospital. Subjects were eligible to participate when they were aged between 18 and 50 years, healthy and had no history of thyroid disease or renal insufficiency. Exclusion criteria included: body mass index (BMI) above 30 kg/m^2^, current smoker, structural alcohol intake >3 units/day, any acute or chronic illness including diabetes mellitus, inflammatory disease, cardiac or respiratory conditions, use of medication, use of supplements that contain large quantities of iodine, subjects who had undergone imaging studies using iodine containing contrast in the last three months, subjects who have taken part in any drug trial within two months prior to start of this study. Eligibility was verified by interview and laboratory testing for TSH and creatinine. The Medical Ethics Committee of the Radboud University Medical Center, Nijmegen, The Netherlands approved the study protocol, and all participants provided written informed consent. Experiments were conducted In the Radboud University Medical Center Nijmegen, The Netherlands between October 2016 and May 2017, in accordance with the principles expressed in the Declaration of Helsinki. The trial was registered in the Dutch National Trial Register (NTR 5853, registered on 10 May, 2016).

### Experimental design

Participants were randomly subjected to metformin or caloric restriction, superposed on a iodine restricted diet. A 4-weeks period with iodine restriction only served as reference. For this proof-of principle pilot study we aimed to include at least seven subjects per intervention group. Subjects who were enrolled after screening received a diary to register daily caloric intake and an estimate of physical activity factor (PAL) according to the Dutch Health Council guidelines^35^. Daily energy need was estimated using the Harris-Benedict equation^36^ for daily resting energy need. Total Energy Expenditure was calculated by multiplying daily resting energy need with the recorded PAL, and lean body mass using the equation described by Hume^37^. Subjects were instructed to follow iodine restriction two weeks prior to baseline measurements, until the end of the study. Supplementary Table 1 states the instructions for the iodine restriction.

After two weeks of iodine restriction, baseline measurements were performed. Blood was drawn to measure serum levels of TSH (s-TSH), free thyroxine (s-fT4), 3,5,3′ triiodothyronine (s-T3) and reverse triiodothyronine (s-rT3). Subjects received 5 MBq of iodine-123 (I-123) orally, and 4, 24 and 48 hours after ingestion I-123 uptake was measured with an uptake probe (thallium-doped sodium iodide (NaI (TL)) crystal (Unispec multi channel analyzer, Canberra Industries inc., CT, USA)). Thyroid I-123 uptake was calculated as percentage of total administered I-123 using the following formula: Uptake (%) = (C_thyroid_ − C_blanc_)/(C_calibration_ − C_bkg_), with C_thyroid_ = number of counts over thyroid; C_blanc_ = number of counts measured at blanc; C_calibration_ = number of counts administered, measured at the calibration source; C_bkg_ = number of counts measured at research location (background radiation).

Next, subjects were randomly assigned to the metformin or hypocaloric diet group using simple block randomization by Y.S. Subjects assigned to the metformin group received metformin (Metformin HCL, Centrafarm B.V., Etten-Leur, The Netherlands) for two weeks, (starting with 500 mg/day, increasing up until 2000 mg/day in the last week). Subjects in the diet group received a meal plan for two weeks for a hypocaloric diet of ~1500 kilocalories/day (an estimated caloric restriction of ~40%), with a high fat (50%), low carbohydrate (30%) content. After two weeks, measurements of serum TSH, fT4, T3, rT3, urinary iodine excretion and I-123 uptake were repeated. Additionally, four subjects continued the low iodine diet after the 2-weeks baseline period to verify influence of time on parameters. Primary outcome was defined as significant difference in thyroid I-123 uptake between baseline and post-intervention, whereas secondary outcomes were defined as changes in serum levels of TSH, fT4, T3, rT3 or T3/rT3 and T3/fT4 ratios. Each subject served as his own control. A flow chart for the experimental design of the study is depicted in Supplementary Fig. 1.

Adherence to the iodine restriction was checked at baseline and post intervention with a questionnaire, entries in the intake-diary and a morning spot urine sample. Adherence to the hypocaloric diet was checked by weight measurements and entries in the diary, whereas metformin use was checked by performing pill counts and checking the diary.

### Laboratory measurements

Levels of s-TSH were directly measured after blood withdrawal using a sandwich Electro Chemi Luminescence Immuno Assay (ECLIA) on a random-access assay system Modular Roche Diagnostic (Roche Diagnostics, Rotkreuz, Switzerland, reference interval 0.27–4.2  mU/L). s-fT4 and s-T3 levels were measured with a competitive Electro Chemi Luminescence Immuno Assay (ECLIA) on a random-access assay system (Roche Diagnostics), reference intervals for s-fT4 and s-T3: 10.0–23.0  pmol/L respectively 1.2–3.1 nmol/L. s-rT3 were determined at a later time point in serum samples stored at −80 °C using an in-house Radioimmunoassay (RIA) at the Erasmus Medical Center Rotterdam, The Netherlands as previously described^38^. Morning urine was stored at −80 °C to measure urinary iodine excretion and creatinine after completion of the study as described previously^39^. Briefly, the urine iodine concentration was measured with the Sandell-Kolthoff reaction method, using a Peltier Thermal Cycler (PTC-200) for the heating and cooling process. The reference interval for urinary iodine excretion was 0.2–0.4 µmol/L. Serum and urinary creatinine concentration were measured by an enzymatic assay (Roche) on a Cobas C6000 anlyzer (Roche Diagnostics). Reference interval for serum creatinine concentration was 60–110 µmol/L, the reference value for urinary creatinine concentrations was < 20 mmol/24 hours.

### Statistics

Statistical analysis was performed by IBM SPSS statistics 22.0 software (NY, USA) and figures were made in Graphpad Prism 5 (CA, USA). Normality was tested using D’Agostino and Pearson omnibus normality test. Continuous variables are expressed as means ± standard error of mean (SEM)/standard deviation (SD) or median with interquartile range (IQR) as indicated in the figure legend. Values for s-TSH showed a skewed distribution and were therefore log-transformed. Regarding I-123 uptake measurements, the percentage of change in I-123 uptake after intervention compared to baseline was calculated as well.

Analysis was performed per protocol. Differences before and after intervention within each group were analyzed using paired t-test for parametric variables, or the Wilcoxon matched-pairs signed rank test for non-parametric variables. Differences between the two groups were analyzed using two-tailed paired t-test for parametric variables, or the Wilcoxon matched-pairs signed rank test for non-parametric variables. The level of significance was defined as p-values below 0.05.

## Supplementary information

Supplemental data

## Data Availability

All data generated during and/or analyzed during the current study are available from the corresponding author on reasonable request.

## References

[1] Bahn Chair RS (2011). Hyperthyroidism and other causes of thyrotoxicosis: management guidelines of the American Thyroid Association and American Association of Clinical Endocrinologists. Thyroid.

[2] Haugen, B. R. M. *et al*. 2015 American Thyroid Association Management Guidelines for Adult Patients with Thyroid Nodules and Differentiated Thyroid Cancer. *Thyroid*, 10.1089/thy.2015.0020 (2015).

[3] Dai G, Levy O, Carrasco N (1996). Cloning and characterization of the thyroid iodide transporter. Nature.

[4] Kogai T, Brent GA (2012). The sodium iodide symporter (NIS): regulation and approaches to targeting for cancer therapeutics. Pharmacology & therapeutics.

[5] Portulano C, Paroder-Belenitsky M, Carrasco N (2014). The Na+/I− symporter (NIS): mechanism and medical impact. Endocr Rev.

[6] Abdulrahman RM (2014). Impact of Metformin and compound C on NIS expression and iodine uptake *in vitro* and *in vivo*: a role for CRE in AMPK modulation of thyroid function. Thyroid.

[7] Andrade BM (2011). A novel role for AMP-kinase in the regulation of the Na+/I− symporter and iodide uptake in the rat thyroid gland. Am J Physiol Cell Physiol.

[8] Cazarin JM, Andrade BM, Carvalho DP (2014). AMP-activated protein kinase activation leads to lysome-mediated NA(+)/I(−)-symporter protein degradation in rat thyroid cells. Hormone and metabolic research = Hormon- und Stoffwechselforschung = Hormones et metabolisme.

[9] Hardie DG (2011). AMP-activated protein kinase: an energy sensor that regulates all aspects of cell function. Genes & development.

[10] Berkowitz SA (2014). Initial choice of oral glucose-lowering medication for diabetes mellitus: a patient-centered comparative effectiveness study. JAMA internal medicine.

[11] Zhou G (2001). Role of AMP-activated protein kinase in mechanism of metformin action. The Journal of clinical investigation.

[12] Pappa T, Alevizaki M (2013). Metformin and thyroid: an update. European thyroid journal.

[13] Han B (2015). Metformin inhibits thyroid cancer cell growth, migration, and EMT through the mTOR pathway. Tumour biology: the journal of the International Society for Oncodevelopmental Biology and Medicine.

[14] Jang EK (2015). Metformin Is Associated with a Favorable Outcome in Diabetic Patients with Cervical Lymph Node Metastasis of Differentiated Thyroid Cancer. European Thyroid Journal.

[15] Klubo-Gwiezdzinska J (2013). Treatment with metformin is associated with higher remission rate in diabetic patients with thyroid cancer. The Journal of clinical endocrinology and metabolism.

[16] Chow SM (2006). Health-related quality-of-life study in patients with carcinoma of the thyroid after thyroxine withdrawal for whole body scanning. The Laryngoscope.

[17] Lim DJ (2015). Differences in physicians’ and patients’ perception of acute hypothyroid symptoms induced by thyroid hormone withdrawal in thyroid cancer patients: a multicenter survey in Korea. European thyroid journal.

[18] Ju DL (2016). Dietary evaluation of a low-iodine diet in Korean thyroid cancer patients preparing for radioactive iodine therapy in an iodine-rich region. Nutrition research and practice.

[19] Boelen A, Wiersinga WM, Fliers E (2008). Fasting-induced changes in the hypothalamus-pituitary-thyroid axis. Thyroid.

[20] Danforth E (1979). Dietary-induced alterations in thyroid hormone metabolism during overnutrition. The Journal of clinical investigation.

[21] Heemstra KA (2009). Type 2 iodothyronine deiodinase in skeletal muscle: effects of hypothyroidism and fasting. The Journal of clinical endocrinology and metabolism.

[22] Spaulding SW, Chopra IJ, Sherwin RS, Lyall SS (1976). Effect of caloric restriction and dietary composition of serum T3 and reverse T3 in man. The Journal of clinical endocrinology and metabolism.

[23] Velthuis-te Wierik EJ, Westerterp KR, van den Berg H (1995). Impact of a moderately energy-restricted diet on energy metabolism and body composition in non-obese men. International journal of obesity and related metabolic disorders: journal of the International Association for the Study of Obesity.

[24] Burger AG, O’Connell M, Scheidegger K, Woo R, Danforth E (1987). Monodeiodination of triiodothyronine and reverse triiodothyronine during low and high calorie diets. The Journal of clinical endocrinology and metabolism.

[25] Iacobellis G, Ribaudo MC, Zappaterreno A, Iannucci CV, Leonetti F (2005). Relationship of thyroid function with body mass index, leptin, insulin sensitivity and adiponectin in euthyroid obese women. Clin Endocrinol (Oxf).

[26] Rosenbaum M, Hirsch J, Murphy E, Leibel RL (2000). Effects of changes in body weight on carbohydrate metabolism, catecholamine excretion, and thyroid function. The American journal of clinical nutrition.

[27] Peeters RP (2003). Reduced activation and increased inactivation of thyroid hormone in tissues of critically ill patients. The Journal of clinical endocrinology and metabolism.

[28] Peeters RP (2005). Serum 3,3′,5′-triiodothyronine (rT3) and 3,5,3′-triiodothyronine/rT3 are prognostic markers in critically ill patients and are associated with postmortem tissue deiodinase activities. The Journal of clinical endocrinology and metabolism.

[29] Krysiak R, Szkrobka W, Okopien B (2018). Sex-Dependent Effect of Metformin on Serum Prolactin Levels In Hyperprolactinemic Patients With Type 2 Diabetes: A Pilot Study. Experimental and clinical endocrinology & diabetes: official journal, German Society of Endocrinology [and] German Diabetes Association.

[30] Chatzitomaris A (2017). Thyroid Allostasis-Adaptive Responses of Thyrotropic Feedback Control to Conditions of Strain, Stress, and Developmental Programming. Frontiers in endocrinology.

[31] Roef G (2012). Body composition and metabolic parameters are associated with variation in thyroid hormone levels among euthyroid young men. European journal of endocrinology.

[32] Draznin B, Wang C, Adochio R, Leitner JW, Cornier MA (2012). Effect of dietary macronutrient composition on AMPK and SIRT1 expression and activity in human skeletal muscle. Hormone and metabolic research = Hormon- und Stoffwechselforschung = Hormones et metabolisme.

[33] Musi N (2002). Metformin increases AMP-activated protein kinase activity in skeletal muscle of subjects with type 2 diabetes. Diabetes.

[34] Vidal AP (2013). AMP-activated protein kinase signaling is upregulated in papillary thyroid cancer. European journal of endocrinology.

[35] Gezondheidsraad. Vol. A06/08 (Den Haag., 2006).

[36] Roza AM, Shizgal HM (1984). The Harris Benedict equation reevaluated: resting energy requirements and the body cell mass. The American journal of clinical nutrition.

[37] Hume R (1966). Prediction of lean body mass from height and weight. Journal of clinical pathology.

[38] Visser TJ, Docter R, Hennemann G (1977). Radioimmunoassay of reverse tri-iodothyronine. The Journal of endocrinology.

[39] van de Ven AC (2014). Longitudinal trends in thyroid function in relation to iodine intake: ongoing changes of thyroid function despite adequate current iodine status. European journal of endocrinology.

