# Supplementary material for: The Influence of Energy Depletion by Metformin or Hypocaloric Diet on Thyroid Iodine Uptake in Healthy Volunteers: a Randomized Trial
Source: Sci Rep. 2019 Apr 1;9:5396. doi: 10.1038/s41598-019-41997-2 (PMC6443645; doi:10.1038/s41598-019-41997-2)
Supplement: Supplementary file 1 — Supplemental data [file 41598_2019_41997_MOESM1_ESM.pdf]

Supplemental file for

The Influence of Energy Depletion by Metformin or Hypocaloric Diet on Thyroid Iodine Uptake in Healthy Volunteers: a Randomized Trial

Yvette J.E. Sloot M.D., Marcel J.R. Janssen M.D. PhD., Antonius E. van Herwaarden PhD, Robin P. Peeters M.D. PhD, Romana T. Netea-Maier M.D. PhD, Johannes W.A. Smit M.D. PhD.

Supplementary table 1 Instructions for the iodine restriction during the study

Products that were not allowed	Other recommendations
Seafood, including fish, seaweed and shellfish,	When adding salt to a meal, use non-iodized salt
Red food color E-127	Reduce use of iodide containing soaps , ointments etc.
Vitamine supplements containing iodide	
Cough syrup, cough lozenges (Stophoest ®) etc.	
Homeopathic drugs containing iodide	

Supplementary table 2 Effect of hypocaloric dieting and metformin on weight, BMI and corrected urinary iodine excretion

	**Hypocaloric diet n=7**		**Metformin n=8**	
	**Baseline**	**Post intervention**	**Baseline**	**Post intervention**
Weight (kg)	79.1 (11.7)	77.4 (12.4)	73.7 (6.9)	72.9 (6.7)
Nett weight loss (kg)		-1.8 (2.4)		-0.8 (1.4)
BMI (kg/m^2)	22.8 (2.4)	22.2 (2.3)	22.6 (2.2)	22.4 (2.3)
Corrected urinary iodine excretion (ug I/g Cr).	50.2 (18.1 - 88.2)	88.2 (38.3 -119.8)	71.0 (33.1 - 133.9)	73.65 (42.3 - 69.5)

Notes: Values for weight, weight loss and BMI are depicted as mean ± S.D., values for corrected urinary iodine excretion are depicted as median ± interquartile range.

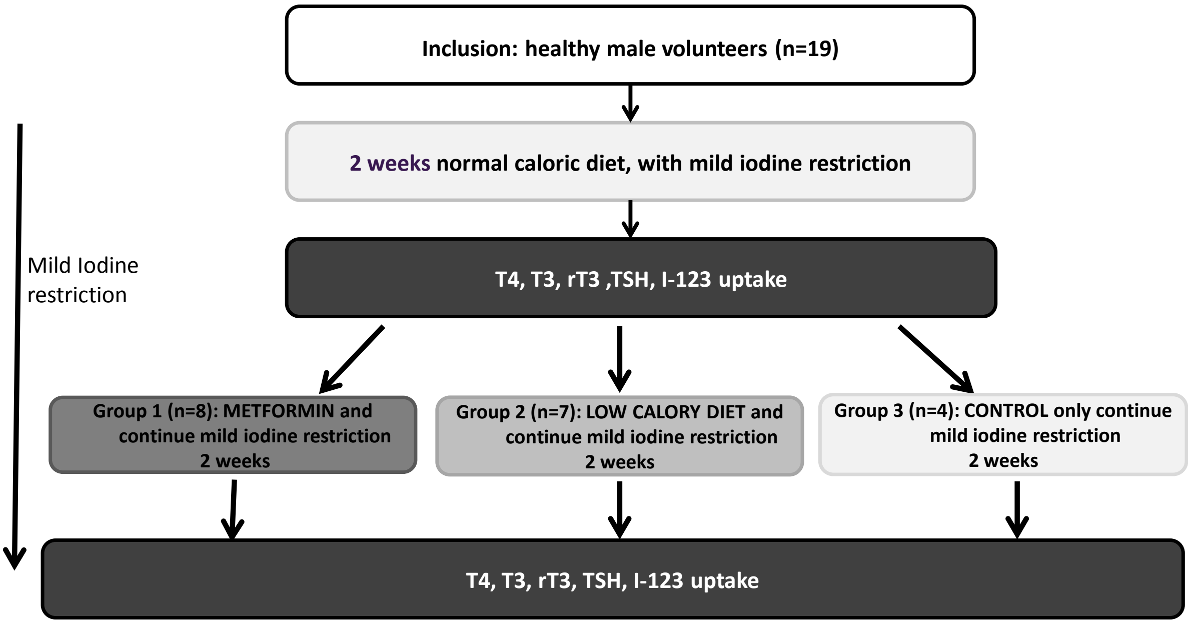

Supplementary figure 1 Flow chart for the experimental design of the study.
